# Supplementary material for: 5α-cyprinol sulfate, a bile salt from fish, induces diel vertical migration in Daphnia
Source: eLife. 2019 May 2;8:e44791. doi: 10.7554/eLife.44791 (PMC6559785; doi:10.7554/eLife.44791)
Supplement: Figure 1—source data 2. — Statistical analysis of mean daytime residence depth of Daphnia magna in response to different HPLC-fractions (Frac 1 - Frac 6) of extracted fish incubation water (EFI) as shown in Figure 1B. Significantly different pairwise comparisons are given in red, n.s.: not significant. [file elife-44791-fig1-data2.docx]

**Figure 1—source data 2.** **Response of *Daphnia* to HPLC fractions.** Statistical analysis of mean daytime residence depth of *Daphnia magna* in response to different HPLC-fractions (Frac 1 - Frac 6) of extracted fish incubation water (EFI) as shown in Figure 1B. Significantly different pairwise comparisons are given in red, n.s.: not significant.

|  | Response to HPLC fractions of extracted fish incubation water (EFI) | | | | | | | |
| --- | --- | --- | --- | --- | --- | --- | --- | --- |
|  | One-way ANOVA, F_7,32_=2233, p<0.0001. | | | | | | | |
|  | Tukey's HSD, pairwise comparisons | | | | | | | |
|  | Control | EFI | Frac 1 | Frac 2 | Frac 3 | Frac 4 | Frac 5 | Frac 6 |
| Control |  | <0.0001 | n.s. | n.s. | <0.01 | n.s. | n.s. | n.s. |
| EFI |  |  | <0.0001 | <0.0001 | n.s. | <0.001 | <0.0001 | <0.0001 |
| Frac 1 |  |  |  | n.s. | <0.05 | n.s. | n.s. | n.s. |
| Frac 2 |  |  |  |  | <0.05 | n.s. | n.s. | n.s. |
| Frac 3 |  |  |  |  |  | n.s. | <0.01 | <0.01 |
| Frac 4 |  |  |  |  |  |  | n.s. | n.s. |
| Frac 5 |  |  |  |  |  |  |  | n.s. |
| Frac 6 |  |  |  |  |  |  |  |  |
