# Supplementary material for: 5α-cyprinol sulfate, a bile salt from fish, induces diel vertical migration in Daphnia
Source: eLife. 2019 May 2;8:e44791. doi: 10.7554/eLife.44791 (PMC6559785; doi:10.7554/eLife.44791)
Supplement: Figure 3—figure supplement 2—source data 1. — Statistical analysis of mean daytime residence depth of Daphnia magna in response to extracted fish incubation water (EFI) and to extract of carp bile and trout bile as shown in Figure 3—figure supplement 2. Significantly different pairwise comparisons are given in red, n.s.: not significant. [file elife-44791-fig3-figsupp2-data1.docx]

**Figure 3—figure supplement 2—source data 1.** **Response of *Daphnia* to extracts of fish incubation water and fish bile.** Statistical analysis of mean daytime residence depth of *Daphnia magna* in response to extracted fish incubation water (EFI) and to extract of carp bile and trout bile as shown in Figure 3—figure supplement 2. Significantly different pairwise comparisons are given in red, n.s.: not significant.

|  | Behavioral response of *Daphnia magna* to extract of fish incubation water (EFI) and to extract of carp and trout bile | | | | | |
| --- | --- | --- | --- | --- | --- | --- |
|  | One-way ANOVA, F_5,18_=6.127, p<0.01. | | | | | |
|  | Tukey's HSD, pairwise comparisons | | | | | |
|  | Control | EFI | carp bile [1 nM 5α-CPS ] | carp bile [2 nM 5α-CPS ] | trout bile [2.5 nM 5α-CPS] | trout bile [25 nM 5α-CPS] |
| Control |  | <0.01 | <0.01 | <0.05 | n.s. | <0.05 |
| EFI |  |  | n.s. | n.s. | n.s. | n.s. |
| carp bile [1nM CPS] |  |  |  | n.s. | n.s. | n.s. |
| carp bile [2nM CPS] |  |  |  |  | n.s. | n.s. |
| trout bile [2.5nM CPS] |  |  |  |  |  | n.s. |
| trout bile [25 nM CPS] |  |  |  |  |  |  |
