# Supplementary material for: 5α-cyprinol sulfate, a bile salt from fish, induces diel vertical migration in Daphnia
Source: eLife. 2019 May 2;8:e44791. doi: 10.7554/eLife.44791 (PMC6559785; doi:10.7554/eLife.44791)
Supplement: Figure 4—source data 2. — Statistical analysis of mean daytime residence depth of Daphnia magna in response to different concentrations of 5α-cyprinol sulfate (5α-CPS) as shown in Figure 4A. Significantly different pairwise comparisons are given in red, n.s.: not significant. [file elife-44791-fig4-data2.docx]

**Figure 4—source data 2.** **Response of *Daphnia* to 5α-cyprinol sulfate.** Statistical analysis of mean daytime residence depth of *Daphnia magna* in response to different concentrations of 5α-cyprinol sulfate (5α-CPS) as shown in Figure 4**A**. Significantly different pairwise comparisons are given in red, n.s.: not significant.

|  | Response to different concentrations of 5α-cyprinol sulfate (5α-CPS) | | | | | | |
| --- | --- | --- | --- | --- | --- | --- | --- |
|  | One-way ANOVA, F_6,23_=24.829, p<0.0001. | | | | | | |
|  | Tukey's HSD, pairwise comparisons | | | | | | |
|  | Control | EFI | 1 pM 5α-CPS | 10 pM 5α-CPS | 107 pM CPS | 1070 pM 5α-CPS | 10700 pM 5α-CPS |
| Control |  | <0.0001 | n.s. | n.s. | <0.0001 | <0.0001 | <0.001 |
| EFI |  |  | <0.0001 | <0.0001 | n.s. | n.s. | n.s. |
| 1 pM CPS |  |  |  | n.s. | <0.0001 | <0.0001 | <0.0001 |
| 10 pM CPS |  |  |  |  | <0.0001 | <0.0001 | <0.0001 |
| 107 pM CPS |  |  |  |  |  | n.s. | n.s. |
| 1070 pM CPS |  |  |  |  |  |  | n.s. |
| 10700 pM CPS |  |  |  |  |  |  |  |
