# Supplementary material for: 5α-cyprinol sulfate, a bile salt from fish, induces diel vertical migration in Daphnia
Source: eLife. 2019 May 2;8:e44791. doi: 10.7554/eLife.44791 (PMC6559785; doi:10.7554/eLife.44791)
Supplement: Figure 4—source data 3. — Statistical analysis of mean daytime residence depth of Daphnia magna in response to different concentrations of taurochenodeoxycholic acid (TCDCA) as shown in Figure 4B. Significantly different pairwise comparisons are given in red, n.s.: not significant. [file elife-44791-fig4-data3.docx]

**Figure 4—source data 3.** **Response of *Daphnia* to taurochenodeoxycholic acid (TCDCA).** Statistical analysis of mean daytime residence depth of *Daphnia magna* in response to different concentrations of taurochenodeoxycholic acid (TCDCA) as shown in Figure 4**B**. Significantly different pairwise comparisons are given in red, n.s.: not significant.

|  | Response to different concentrations of taurochenodeoxycholic acid (TCDCA) | | | | | | |  | |  | |
| --- | --- | --- | --- | --- | --- | --- | --- | --- | --- | --- | --- |
|  | One-way ANOVA, F_8,25_=9.182, p<0.0001. | | | | | | |  | |  | |
|  | Tukey's HSD, pairwise comparisons | | | | | | |  | |  | |
|  | Control | EFI | 19 pM TCDCA | 1.9x10^2^ pM TCDCA | 1.9x10^3^ pM TCDCA | 1.9x10^4^ pM TCDCA | 1.9x10^5^ pM TCDCA | | 1.9x10^6^ pM TCDCA | | 1.9x10^7^ pM TCDCA |
| Control |  | n.s. | n.s. | n.s. | n.s. | n.s. | n.s. | | n.s. | | <0.01 |
| EFI |  |  | <0.001 | <0.001 | <0.001 | <0.001 | <0.01 | | n.s. | | n.s. |
| 19 pM TCDCA |  |  |  | n.s. | n.s. | n.s. | n.s. | | n.s. | | n.s. |
| n1.9x10^2^ pM TCDCA |  |  |  |  | n.s. | n.s. | n.s. | | n.s. | | <0.01 |
| 1.9x10^3^ pM TCDCA |  |  |  |  |  | n.s. | n.s. | | n.s. | | <0.01 |
| 1.9x10^4^ pM TCDCA |  |  |  |  |  |  | n.s. | | n.s. | | <0.01 |
| 1.9x10^5^ pM TCDCA |  |  |  |  |  |  |  | | n.s. | | n.s. |
| 1.9x10^6^ pM TCDCA |  |  |  |  |  |  |  | |  | | n.s. |
| 1.9x10^7^ pM TCDCA |  |  |  |  |  |  |  | |  | |  |
